# A Study on the Use of Nebulized Glycopyrronium in Hospitalized Patients With Exacerbations of Obstructive Airway Disease

**DOI:** 10.7759/cureus.104894

**Published:** 2026-03-09

**Authors:** Arjun Khanna, Abhishek Tandon, Lakshmi Narasimhan R

**Affiliations:** 1 Department of Pulmonology Medicine, School of Medicine, Amrita Vishwa Vidyapeetham, Faridabad, IND; 2 Department of Respiratory and Sleep Medicine, Medanta, Lucknow, IND; 3 Department of Pulmonology, Manipal Hospitals, Mysore, IND

**Keywords:** acute exacerbation of chronic obstructive pulmonary disease, bronchodilator, copd, glycopyrronium, long-acting muscarinic antagonist (lama), nebulization, obstructive airway diseases

## Abstract

Introduction

Acute exacerbations of obstructive airway diseases (OAD) are commonly encountered respiratory conditions that often require hospitalization and intensive bronchodilator therapy. While short-acting β2-agonists (SABAs) and muscarinic antagonists (SAMAs) are commonly used, nebulized glycopyrronium, a long-acting muscarinic antagonist (LAMA), may offer better outcomes.

Methods

This was a multicenter, retrospective study done across 278 sites to assess the overall utilization and effectiveness of nebulized glycopyrronium in managing exacerbations in hospitalized patients with obstructive airway disease in a real-world Indian setting. Medical records of hospitalized patients were analyzed, where nebulized glycopyrronium was used in the management of exacerbation for information regarding utilization and effectiveness. Outcomes included the length of intensive care unit (ICU) stay, ward stay, oxygen requirement, ventilation support, adverse events, and mortality.

Results

A total of 2,780 medical records were analyzed across multiple tertiary care centers. The analyzed population had a mean age of 59.56 years (±9.72). Nebulized glycopyrronium was administered as combination therapy in 1,306 (48.44%) patients and as monotherapy in 1,390 (51.56%) patients. The majority of the patients (2,336, 90.61%) received twice-a-day (BID) dosing. The mean duration of glycopyrronium therapy was 13.45 days. A total of 68 adverse events were reported, most of which were mild and self-limiting. Dry mouth was the most frequent complaint (13, 0.46%), followed by dizziness and transient headache. Mortality was observed in 42 (1.52%) of the treated population, with no direct attribution to glycopyrronium therapy.

Conclusion

Nebulized glycopyrronium is an effective therapy in hospitalized patients with obstructive airway disease, offering faster recovery and reduced oxygen dependency.

## Introduction

The acute exacerbation of asthma and chronic obstructive pulmonary disease (COPD) are commonly encountered respiratory conditions requiring immediate medical evaluation and treatment. These conditions cause rapid deterioration in lung function and can significantly impair the quality of life [1]. An Indian study reported that the in-hospital mortality of COPD related to acute exacerbations is 12%, while one-year mortality varied from 22% to 43%, and two-year mortality varied from 36% to 49%, thus highlighting the need for the prompt and effective management of exacerbations [2].

Acute exacerbations often necessitate hospitalization and aggressive bronchodilator therapy. Traditionally, short-acting β2-agonists (SABAs) and short-acting muscarinic antagonists (SAMAs) have been the mainstay of treatment. However, frequent dosing and cardiovascular side effects limit their utility in severe cases [3]. However, glycopyrronium, a long-acting muscarinic antagonist (LAMA), has a rapid onset (showing statistically significant bronchodilation within five and 15 minutes post-dose), resulting in better early bronchodilation during the first four hours post-dose, with an acceptable safety and tolerability profile [4].

However, there are no data on the use of nebulized glycopyrronium in the acute setting. The objective of this study was to assess the overall utilization and efficacy of nebulized glycopyrronium in hospitalized patients due to exacerbations of obstructive airway disease (OAD).

## Materials and methods

Study design

This was a multicentric, retrospective study conducted at 278 sites across India. A predesigned structured proforma was used for this study to collect information from the medical records of the patients prescribed nebulized glycopyrronium by their treating physician. These data were entered into an electronic case report form (eCRF). All medical records were fully de-identified prior to data extraction and entry into the eCRF to ensure patient confidentiality. The study was initiated after approval from the Good Society for Ethical Research-Independent Ethics Committee (approval number: GSER/2024/BMR-AP/119). The data collection for this study was conducted over a six‑month period. The study was conducted in accordance with applicable regulations, good clinical practice (GCP), and standard operating procedures

Study participants

Medical records of adult patients who were prescribed nebulized glycopyrronium, either as monotherapy or in combination with inhaled corticosteroids (ICS)/long-acting beta‑agonist (LABA), for acute exacerbation related to obstructive airway disease (OAD) in the emergency room or intensive care unit (ICU) starting from day 1 were included in the study.

Study endpoints

The primary endpoints of this study focused on evaluating the overall utilization of nebulized glycopyrronium among hospitalized patients. Specifically, the analysis assessed its preference as monotherapy versus combination therapy, the duration of administration, and the pattern of concomitant medication use. Secondary endpoints included the frequency of rescue medication use, systemic corticosteroid administration, and the proportion of patients requiring supplemental oxygen therapy or ventilatory support, ICU stay, and ward stay. Safety endpoints were also examined, encompassing the incidence and nature of adverse events, as well as overall mortality.

Statistical analyses

Descriptive statistics were used to summarize all study variables. Categorical data were presented as absolute numbers and percentages (N and %), and continuous variables were expressed as mean ± standard deviation (SD). Data extraction and cleaning were performed in Microsoft Excel (Microsoft Corp., Redmond, WA), and statistical analyses were conducted using IBM SPSS Statistics version 26.0 (IBM Corp., Armonk, NY). All analyses were performed on available data without imputation for missing values.

## Results

Demographics and baseline characteristics

Medical records of 2,780 patients were available for analysis. The mean age of the enrolled patients was 59.56 years (±9.72), with a notable male predominance (2,103, 75.64%). The mean duration of OAD among patients was 7.92 ± 7 years (n = 2,680). A significant proportion of patients (1,609, 57.9%) presented with a prior history of exacerbations, and 1,213 (43.62%) had previously been hospitalized for OAD-related complications.

Diagnosis summary and medical history

Among the enrolled patients, almost two-thirds (1,804, 65.12%) were diagnosed with COPD, followed by asthma (523, 18.88%), asthma-COPD overlap syndrome (ACOS) in 422 (15.23%), and other respiratory indications in 21 (0.77%) cases (Table 1). Hypertension (1,340, 48.20%) and diabetes (1,153, 41.47%) were the most common comorbidities in hospitalized patients with exacerbation (Table 2).

**Table 1:** Comorbidities in hospitalized patients with exacerbation of obstructive airway disease Values are presented as the number of patients (N) with corresponding percentages (%) for each comorbidity listed OSA, obstructive sleep apnea; OSAHS, obstructive sleep apnea-hypopnea syndrome; TB, tuberculosis; SDH, subdural hematoma; N, not available

Parameter	N (%)	
Hypertension	1,340 (48.20%)	
Diabetes	1,153 (41.47%)	
Coronary artery disease	207 (7.45%)	
Osteoporosis	200 (7.19%)	
Arrhythmia	121 (4.35%)	
Atrial fibrillation	65 (2.34%)	
Others	279 (10.03%)	
Allergic rhinitis		20 (0.72%)
Ankylosing Spondylitis		1 (0.04%)
Cardiovascular disease		1 (0.04%)
Cerebrovascular disease with ischemic stroke		1 (0.04%)
Fatigue		1 (0.04%)
NA		1 (0.04%)
OSA		1 (0.04%)
OSAHS		1 (0.04%)
Obesity		1 (0.04%)
Old TB		1 (0.04%)
Rheumatoid arthritis		1 (0.04%)
SDH post burr hole surgery		1 (0.04%)
Shortness of breathing		1 (0.04%)
Tachycardia		1 (0.04%)

**Table 2:** Diagnosis summary Data are presented as N (%). Percentages were calculated using the respective column‑header denominator COPD, chronic obstructive pulmonary disease; ACOS, asthma-COPD overlap syndrome; TB, tuberculosis

Parameter (N = 2,770)	Categories	N (%)
Diagnosis	COPD	1,804 (65.12%)
	Asthma	523 (18.88%)
	ACOS	422 (15.23%)
	Others	21 (0.77%)
Others	COPD with post TB sequelae	10 (0.37%)
	Chronic bronchitis	2 (0.074%)
	Emphysema	2 (0.074%)
	Interstitial lung disease	2(0.074%)
	Combined pulmonary fibrosis and emphysema (CPFE)	1 (0.037%)
	Bronchiectasis	1 (0.037%)
	Pneumonia with COPD exacerbation	1 (0.037%)
	Allergic bronchopulmonary aspergillosis (ABPA)	1 (0.037%)
	Bilateral lower zone consolidation	1 (0.037%)

Utilization of nebulized glycopyrronium

Nebulized glycopyrronium was administered as a combination in 1,306 (48.44%) patients and as monotherapy in 1,390 (51.56%) patients. Twice-a-day (BID) dosing of nebulized glycopyrronium was given to 2,336 (90.61%) patients, whereas once-a-day (OD) dosing was given to 242 (9.39%) patients. It was administered for a mean duration of 13.45 days. Nebulized glycopyrronium with nebulized formoterol-budesonide as a combination was prescribed to 445 (16%) patients on day 1, while nebulized glycopyrronium with formoterol-budesonide + levosalbutamol (SABA) was prescribed to 174 (6.25%) subjects and with a combination of ipratropium-levosalbutamol (SAMA-SABA) in 203 (7.3%) patients on day 1. ICS was prescribed in 622 (22.5%) subjects at day 1 (Table 3).

**Table 3:** Concomitant medications with nebulized glycopyrronium Data are presented as N (%). Percentages were calculated using the respective daily denominator ICS, inhaled corticosteroid; SABA, short‑acting β2‑agonist; SAMA, short‑acting muscarinic antagonist

Combinations	Day 1, n (%)	Day 2, n (%)	Day 3, n (%)	Day 4, n (%)	Day 5, n (%)
Formoterol-budesonide	445 (16)	311 (11.18)	303 (10.89)	283 (10.17)	327 (11.76)
Formoterol-budesonide + levosalbutamol (SABA)	174 (6.25)	122 (4.38)	70 (2.51)	31 (1.11)	31 (1.11)
Formoterol-budesonide + ipratropium (SAMA)	16 (0.57)	6 (0.21)	4 (0.14)	2 (0.07)	1 (0.03)
Formoterol-budesonide + ipratropium-levosalbutamol (SAMA-SABA)	203 (7.3)	109 (3.92)	78 (2.8)	61 (2.19)	90 (3.23)
Ipratropium-levosalbutamol (SAMA-SABA)	119 (4.28)	86 (3.09)	78 (2.8)	62 (2.23)	56 (2.01)
ICS	622 (22.55)	440 (15.83)	428 (15.40)	342 (12.30)	368 (13.25)
Antibiotics	1,091 (39.24)	811 (29.17)	776 (27.91)	684 (24.60)	725 (26.08)

Requirement of supplemental oxygen therapy or ventilation and the average length of ICU and ward stay

High-flow nasal oxygen therapy (HFNO) was required by 627 (22.55%) patients at day 1, which reduced to 224 (8.06%) at day 5, whereas low-flow nasal oxygen (LFNO) was given to 417 (15%) patients at day 1, which reduced to 244 (8.78%) at day 5. Noninvasive ventilation (NIV) was required by 297 (10.68%) patients at day 1, which reduced to 214 (7.70%) patients at day 5. Mechanical ventilation was required by 267 (9.60%) patients at day 1, which reduced to 180 (6.47%) patients at day 5. The average duration of ICU stay was 3.15 days (n = 2,369), while the mean ward stay was 4.96 days (n = 2,501), indicating relatively rapid clinical stabilization and discharge readiness in most patients (Table 4).

**Table 4:** Proportion of patients requiring additional oxygen therapy or ventilation up to day 5 of treatment initiation Data are presented as N (%). Percentages were calculated using the respective column‑header denominator HFNO, high‑flow nasal oxygen; LFNO, low‑flow nasal oxygen; NIV, noninvasive ventilation

Parameter	Categories	N (%)
Requirement of oxygen therapy (HFNO)	Day 1	627 (22.55%)
	Day 2	502 (18.06%)
	Day 3	445 (16.01%)
	Day 4	285 (10.25%)
	Day 5	224 (8.06%)
Requirement of oxygen therapy (LFNO)	Day 1	417 (15.00%)
	Day 2	432 (15.54%)
	Day 3	543 (19.53%)
	Day 4	359 (12.91%)
	Day 5	244 (8.78%)
Requirement of NIV	Day 1	297 (10.68%)
	Day 2	212 (7.63%)
	Day 3	302 (10.86%)
	Day 4	239 (8.60%)
	Day 5	214 (7.70%)
Requirement of mechanical ventilation	Day 1	267 (9.60%)
	Day 2	238 (8.56%)
	Day 3	295 (10.61%)
	Day 4	240 (8.63%)
	Day 5	180 (6.47%)

Adverse events

Adverse events were reported in 68 (2.45%) patients in the study. The most common adverse events were dry mouth and constipation. Mortality was observed in 42 (1.53%) of the study population, with no direct causal link established to glycopyrronium administration (Tables 5, 6).

**Table 5:** At discharge and the average length of ICU and ward stay Data are presented as mean (SD) ICU, intensive care unit; SD, standard deviation

	n	Mean (SD)
Number of days in ICU	2,369	3.15 (2.93)
Number of days in the ward	2,501	4.96 (3.03)

**Table 6:** Summary of adverse events Data are presented as N (%). Percentages were calculated using the respective column‑header denominator GERD: gastroesophageal reflux disease

Parameter	Categories	N (%)
Any adverse event during the treatment?	Yes	68 (2.45%)
	No	2,712 (97.90%)
Adverse events	Dry mouth	13 (0.46%)
	Constipation	6 (0.2%)
	Atrial fibrillation	2 (0.07%)
	Allergy	2 (0.07%)
	Exacerbation	2 (0.07%)
	Anxiety	1 (0.04%)
	GERD	1 (0.04%)
	Mild tachycardia	1 (0.04%)
	More exacerbation	1 (0.04%)
	Tightness in the chest	1 (0.04%)
	Urine retention	1 (0.04%)
Mortality	Yes	42 (1.52%)
	No	2,738 (98.48%)

## Discussion

This large multicentric study provides the real-world utilization of nebulized glycopyrronium in hospitalized patients with acute exacerbations of obstructive airway disease (OAD).

The study included 2,780 patients across multiple centers. The study provides real-world insights into nebulized glycopyrronium utilization patterns, associated oxygen and ventilation support requirements, ICU and ward stay durations, and the spectrum of reported adverse effects.

The demographic and baseline characteristics data reflect patients with a recurrent exacerbation pattern, underscoring the need for effective inpatient management strategies. The diagnosis summary highlights the predominance of COPD in patients hospitalized for OAD. Hypertension and diabetes mellitus emerged as the two most common comorbid conditions, indicating the typical cardiovascular and metabolic burden associated with chronic airway diseases.

The predominance of BID dosing (2,336, 90.61%) reflects clinical confidence in glycopyrronium’s sustained bronchodilator effect. Formoterol-budesonide was most commonly co-administered, along with nebulized glycopyrronium. The frequent co-administration of formoterol-budesonide aligns with current Global Initiative for Chronic Obstructive Lung Disease (GOLD) and Global Initiative for Asthma (GINA) guidelines, supporting the use of triple therapy in high-risk patients.

Our analysis revealed that nebulized glycopyrronium significantly improved clinical outcomes, as seen by a marked decline in oxygen requirement in any form. There was also decreased reliance on SABA rescue inhalers. The average ICU stay and ward stay of subjects were 3.15 and 4.96 days, respectively. In a study conducted by Hasan et al., it was observed that the average length of hospital stay was 5.8 (±1.39) when salbutamol and ipratropium were used in combination [5]. In a matched cohort study by Thomas et al. (2017), patients receiving ipratropium-salbutamol had a mean ICU stay of 4.2 days and hospital stay of 6.1 days, which is notably longer than the 3.15-day ICU stay and 4.96-day ward stay observed in our glycopyrronium cohort [6]. This translates to nebulized glycopyrronium having a shorter or comparable hospital stay compared to SABA and SAMA. Hence, when compared to conventional SABA + SAMA regimens, glycopyrronium demonstrated better clinical outcomes.

The frequent or high‑dose administration of short‑acting β2‑agonists (SABAs) and ipratropium during acute exacerbations can provoke cardiovascular effects within minutes, including tachycardia, tremors, and arrhythmias [7,8]. These risks are amplified in COPD, where baseline cardiovascular comorbidity is common, and are supported by evidence linking higher inpatient SABA exposure to elevated N-terminal pro-brain natriuretic peptide (NT‑proBNP), a biomarker of cardiac stress, in COPD exacerbations [9].

Pharmacologically, glycopyrronium demonstrates several advantages, including bronchodilator onset within 5-30 minutes, sustained action, and low systemic absorption, which translates to fewer anticholinergic adverse effects and negligible central nervous system penetration [10]. In a comparative study evaluating nebulized glycopyrronium versus a salbutamol-ipratropium combination in critically ill, mechanically ventilated patients with COPD, glycopyrronium not only achieved faster bronchodilation but also maintained reduced airway resistance for up to 12 hours [11]. Notably, this extended effect was observed without adverse events such as hypertension, tachycardia, or the desiccation of respiratory secretions, underscoring its potential for both efficacy and safety in the acute care setting.

Our cohort experienced a few adverse events, predominantly dry mouth. Tachycardia and atrial fibrillation were seen in very few patients.

In contrast, a systematic review of 10 clinical trials found no significant improvement in outcomes with higher doses of short-acting β2-agonists (SABAs), but increased doses led to more cardiac side effects without added bronchodilator benefit when used for the management of acute exacerbations [12].

In summary, nebulized glycopyrronium demonstrated effective bronchodilation, reduced oxygen dependency, and shorter hospital stays compared to conventional regimens, with a favorable safety profile. These findings support its role as a reliable inpatient management option for acute exacerbations of OAD.

The limitations of our study were a retrospective design, small sample size, short-term follow-up, and lack of head-to-head comparisons with SABA/SAMA. Additionally, no advanced statistical analyses were performed, as the study was designed primarily as a retrospective assessment rather than an analytical comparative study.

Nevertheless, our real-world data provide valuable insights into an often overlooked intervention, nebulized glycopyrronium therapy during hospital admissions. Future directions should include large, multicenter randomized controlled trials (RCTs) comparing nebulized glycopyrronium directly to SABAs/SAMAs on parameters such as cardiovascular adverse events, the use of corticosteroids, and cost-effectiveness evaluations, particularly focusing on the length of hospital stay, resource use, and rehospitalization rates.

## Conclusions

In this large, multicenter, real-world cohort of hospitalized patients with acute exacerbations of obstructive airway disease, nebulized glycopyrronium was well‑tolerated and consistently associated with meaningful clinical benefits. Treatment led to marked reductions in oxygen and ventilatory support needs, shorter ICU and ward stays compared to published outcomes for SABA-SAMA regimens, and a lower reliance on rescue bronchodilators. Collectively, these results support nebulized glycopyrronium as an effective and safe treatment similar to conventional short-acting bronchodilator-based protocols in the acute inpatient setting, with the potential to enhance recovery and optimize healthcare resource utilization.
